# Nanoparticles and Nanostructured Surface Fabrication for Innovative Cranial and Maxillofacial Surgery

**DOI:** 10.3390/ma13235391

**Published:** 2020-11-27

**Authors:** Simona Cavalu, Iulian Vasile Antoniac, Aurel Mohan, Florian Bodog, Cristian Doicin, Ileana Mates, Mihaela Ulmeanu, Roman Murzac, Augustin Semenescu

**Affiliations:** 1 Faculty of Medicine and Pharmacy, University of Oradea, 10 P-ta 1 Decembrie, 410073 Oradea, Romania; scavalu@uoradea.ro; 2 Faculty of Materials Sciences Engineering, University Politechnica Bucharest, 313 Splaiul Independentei, 060042 Bucharest, Romania; iulian.antoniac@upb.ro (I.V.A.); cristian.doicin@upb.ro (C.D.); ileana-mariana.mates@upb.ro (I.M.); mihaela.ulmeanu@upb.ro (M.U.); roman.murzac@upb.ro (R.M.); 3 Romanian Academy of Scientists, 3 Ilfov, 077190 Bucharest, Romania; 4 Carol Davila University, Emergency Military Hospital, 134 Calea Plevnei, 010825 Bucharest, Romania

**Keywords:** titanium cranioplasty, endoprosthesis, patented solutions

## Abstract

A novel strategy to improve the success of soft and hard tissue integration of titanium implants is the use of nanoparticles coatings made from basically any type of biocompatible substance, which can advantageously enhance the properties of the material, as compared to its similar bulk material. So, most of the physical methods approaches involve the compaction of nanoparticles versus micron-level particles to yield surfaces with nanoscale grain boundaries, simultaneously preserving the chemistry of the surface among different topographies. At the same time, nanoparticles have been known as one of the most effective antibacterial agents and can be used as effective growth inhibitors of various microorganisms as an alternative to antibiotics. In this paper, based on literature research, we present a comprehensive review of the mechanical, physical, and chemical methods for creating nano-structured titanium surfaces along with the main nanoparticles used for the surface modification of titanium implants, the fabrication methods, their main features, and the purpose of use. We also present two patented solutions which involve nanoparticles to be used in cranioplasty, i.e., a cranial endoprosthesis with a sliding system to repair the traumatic defects of the skull, and a cranial implant based on titanium mesh with osteointegrating structures and functional nanoparticles. The main outcomes of the patented solutions are: (a) a novel geometry of the implant that allow both flexible adaptation of the implant to the specific anatomy of the patient and the promotion of regeneration of the bone tissue; (b) porous structure and favorable geometry for the absorption of impregnated active substances and cells proliferation; (c) the new implant model fit 100% on the structure of the cranial defect without inducing mechanical stress; (d) allows all kinds of radiological examinations and rapid osteointegration, along with the patient recover in a shorter time.

## 1. Introduction into Cranioplasty and Craniofacial Reconstruction Using Titanium Meshes and Plates for Major Cranial Defects

As a result of traumatic injuries, congenital deformities, decompressive craniectomies, or bone flap loss due to infections, cranioplasty is performed as a neurosurgery procedure to reshape the imperfections of the skull, with both cosmetic and functional outcomes. This surgical intervention has a long history, dating back to 3000 B.C., according to archeological findings [1]. In 19th century, the use of bone from different donor sites (allographs), such as the ribs or tibia, gained wide population, following the successful case of reimplantation of cranial bone reported by Sir William Macewen in 1885, promoting autografts for cranioplasty [1]. In autograph or autologous cranioplasty, the patient’s own bone flap is stored and reused in during the waiting period, in order to perform bonny closure [2,3]. This technique evolved at the same time with the beginning of modern general anesthesia, but has been abandoned due to high infection and absorption rates. Military conflicts all over the world have proved the necessity for advanced techniques in cranioplasty, requiring immediate treatment with immediate decompressive craniectomy, careful brain debridement, followed by watertight dural repair [2].

The reconstruction of the large cranial vault defects thus continues to be a challenge to craniofacial surgeons and neurosurgeons. Solutions to the challenging problem of repairing skull defects continue to evolve in order to improve patient outcomes. Although autologous bone is still considered the gold standard in cranioplasty, via the replacement of previously removed bone flap, alloplastic material is indicated in difficult situations when autologous bone in unavailable, such as graft infection, resorption, or insufficient donor site availability [4]. Cranial reconstruction using bone grafting techniques, although ideal from the perspective of immunological compatibility, has many disadvantages, such as complex and intense surgical work, time consumption, and high resorption rate [5]. It has been reported that, in the pediatric population, a much higher bone absorption rate was noticed compared to adults when autologous bone was used [6]. Although many different methods have been described in literature, using autologous or alloplastic material, there is still no consensus on which method is better, and continuous multidisciplinary researches are conducted aiming to develop the ideal reconstruction materials. However, there is a general consensus about the features of an ideal material to be used in cranioplasty [7,8,9], including:

- perfect fit into the cranial defect in order to get fast and complete closure
- radiolucent
- resistance to infections
- heat non-deformable
- strengths comparable to that of the surrounding bone
- biomechanical resistance
- easy to manipulate and contour
- inexpensive
- fast application

PMMA (Poly-methylmethacrylate), introduced in the 1960s [10], is a polymerized organic compound of acrylic acid, which can be used either prefabricated, hand-formed, or templated. Being easy to shape, lightweight, and radiolucent, it gained large popularity in recent decades as the most frequently used material for skull reconstruction [10]. Even if the long-term results of PMMA cranioplasty have generally been acceptable, there are some limitations of PMMA, including its exothermic setting reaction (between 70 and 120 °C, which may cause thermal necrosis of healthy bone, and hence additional surgical procedures are required for such saturation with saline solution for protection) and the osteolysis induced by unpolymerized MMA (methylmetacrylate) monomer. Moreover, in vivo fibrous encapsulation may occur as PMMA does not chemically bond to surrounding tissue [11]. However, the possibility of using prefabricate or template patient specific PMMA implants clearly demonstrates their benefits, avoiding the risk of thermal or chemical necrosis [4].

**Figure 1:**
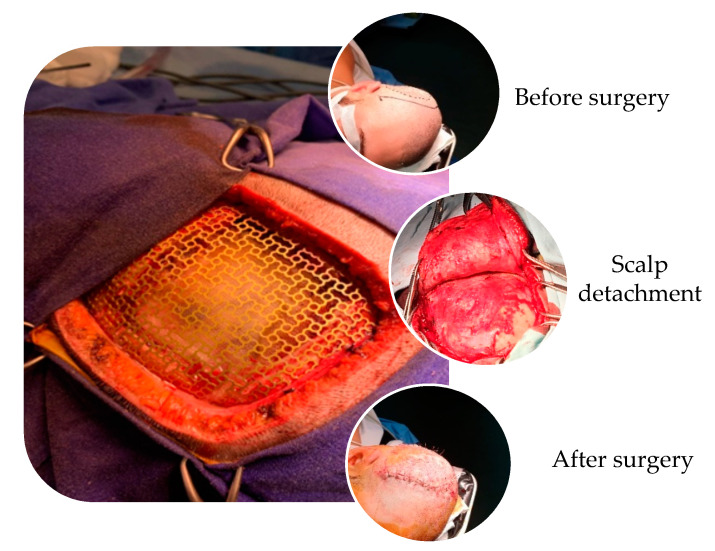
Intraoperatively, surgical details of titanium cranioplasty procedure in the case of a large defect (from private collection of Assoc. Prof. Aurel Mohan).

Titanium cranioplasty using meshes and plates gained large popularity for the reconstruction of moderate to large-sized defects, being readily available and providing strong and malleable mechanical features to be shaped intraoperatively [12]. Unalloyed titanium for implant applications is available in grades 1 to 4, according to a concentration of impurities: 0.2–0.5% Fe and 0.18–0.4 % O_2_ [13]. Titanium is also compatible with magnetic resonance imaging scans [6]. Other favorable properties of titanium mesh for cranioplasty are the stimulation of the bone ingrowth, based on the concept of osteointegration and osteoinduction [14] and its resistance to corrosion from bodily fluids. The standard mesh is a square of 90/90 mm and a thickness of 2.5 mm, which can be easily manipulated by cutting and shaping according to size of the defect. The fixation system is comprised of helically threaded micro-screws, with a screw head of 3 mm in diameter, with at least four points of anchorage [15], but in the case of larger defects, additional fixation points for anchorage are made for optimum geometry. In Figure 1 are presented the surgical details of classical titanium cranioplasty, with the placement and fixation of titanium mesh.

However, adjacent titanium screws for fixation, which is achieved through self-anchoring screws, may cause excessive tension in the titanium mesh, producing titanium mesh edge tilt, temporal muscle compression, and pain [16]. Moreover, titanium cranioplasty (using classical meshes or plates) is associated with a significant risk of complications. There are some large studies in the literature reviewing the complications of titanium cranioplasty which report complication rates as high as 34 % [17] in terms of infection, poor cosmesis, haematoma, headache, and seizures, often necessitating re-operation and plate or mesh removal [17,18]. For example, the study performed by Hill et al. [18] aimed to determine the risk factors of complications following TC (titanium cranioplasty), in a heterogeneous population with various demographic features, taking into account the sizes of cranioplasty. They concluded that infection, seizures, and haemorrhage were the commonest reported complication, while skull defect size was considered to be a determining factor. Instead, the size-related cranioplasty was directly correlated to the hospitalization time. In a similar study conducted by Mukherjee et al. [17], the authors concluded that, besides the size of defect and the traumatic aetiology, the timing of cranioplasty may be important, with late (>12 months) TC associated with a higher rate of complications.

Titanium mesh or plates can be used in cranioplasty either alone or in conjunction with other synthetic materials, such as hydroxyapatite, calcium phosphate, and polyethylene. With commercially available materials such as Medpor Titan [19], a thin sheet of titanium mesh was embedded in a high-density porous polyethylene implant, with better malleability being easily shaped with scissors. This combination provided a fast and effective method for pterional reconstruction after frontotemporal and orbitozygomatic craniotomy, with excellent cosmetic results and patient satisfaction [19]. However, the use of this material in other cranio-anatomical sites or in large defects is not reported. The combination hydroxyapatite-titanium mesh has the advantages of high capacity for osteoconduction and osseointegration, but a high rate of infection was reported when placed in contact with the frontal sinus [20].

Nowadays, one of the strategies adopted in order to overcome the drawbacks and limitations in classical TC is the choice of PEEK (polyetheretherketone) custom implants, prefabricated according to 3D computed tomography and digital modeling of both the cranial defect and the surrounding skeleton, also named “patient-specific implant”. Besides the aesthetic outcomes, there are several advantages to using prefabricated implants for cranioplasty, such as decreased surgical complexity and operative times, which consequently minimize the exposure and risk of contamination [21]. This approach offer a precise fit, along with strength, stiffness, durability, temperature resistance, and radiolucency. However, their high costs might be prohibitive, especially in the case of large defects. Moreover, according to a very recent study performed by Rosinsky et al. [22] infection rates are higher among patients receiving custom implants compared to those receiving titanium meshes, but their long-term complication profile remains to be studied. Therefore, the ideal method for alloplastic cranioplasty with large defects would enable a cost-effective implant choice with the possibility for intraoperative modification, including the cases of tumor resection in which the size of the skull defect is unknown preoperatively, and hence prefabricated implants cannot be used.

This aim of this review is to emphasize the main reasons why titanium mesh is preferred for large skull reconstructions, along with the importance of developing innovative surface structures with a dual benefit: improved osteointegration and enhanced antibacterial activity to reduce the risk of post-surgical infection. Based on literature research, a comprehensive review was conducted on the mechanical, physical, and chemical methods for creating nano-structured titanium surfaces along with the identification of the main nanoparticles used for the surface modification of titanium implants, the fabrication methods, their main features, and the purpose of use.

## 2. The Importance of Nano-Structured Surface on Titanium Implants

**Figure 2:**
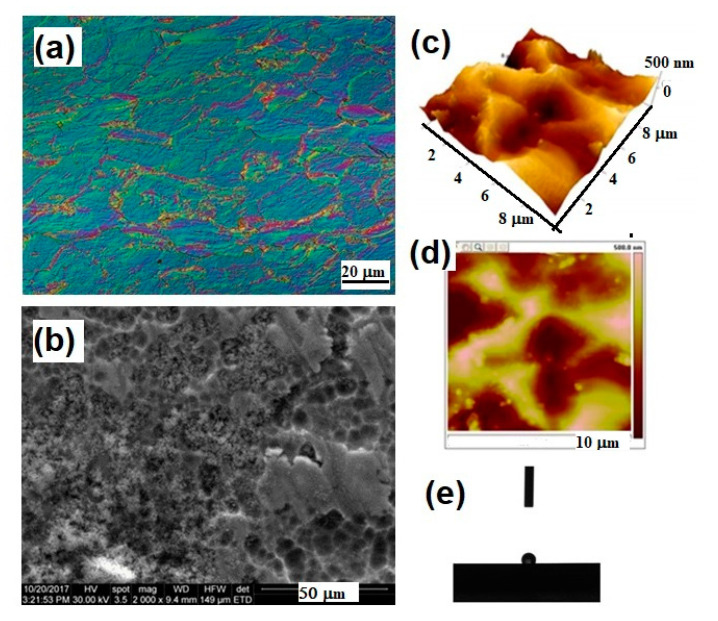
Surface properties of Ti mesh for cranioplasty evidenced by different microscopic techniques: (**a**) light microscopy image in phase contrast, longitudinal section, 500×, Kroll reagent; (**b**) Scanning Electron Microscopy 2000×; (**c**,**d**) 3D and 2D Atomic Force Microscopy images; (**e**) contact angle investigation on the surface of the titanium mesh.

Titanium is the most employed implant material for cranial and maxillofacial applications owing to a specific combination of strength and biocompatibility. Its excellent corrosion resistance in body fluids and mechanical properties that closely match human cortical bone in terms of Young’s modulus (E = 100–110 GPa for pure titanium grade II), resulting in smaller stress shielding compared to other biometals, are the main features taken into account in developing new and versatile implantable devices [23]. Being a bioinert material, it hampers tissue integration, leading to a short lifespan. In Figure 2, metallographic aspects related to the surface analysis of titanium mesh samples are presented using different microscopic techniques.

So, strong efforts have been made to develop innovative, suitable implant surface structures, with the aim to promote osseointegration and to prevent periprosthetic infection. Following the rule of “smaller, faster, cheaper”, nanopatterning with dimensions smaller than 100 nm has encountered clinical applications in orthopedics and traumatology [24,25]. Many studies in the literature have reported that nanometer-controlled titanium surfaces can be fabricated by a variety of surface modifications techniques, including mechanical, chemical, and physical methods, or a combination of these [25,26,27,28,29,30,31,32].

### 2.1. Mechanical, Physical and Chemical a Methods for Creating Nano-Structured Titanium Surfaces

Nowadays, there are commercially available cranial titanium implant whose surface is modified by mechanical methods (either machined or grit blasting), or attrition for obtaining nanophase materials), chemical methods (acid or alkali treatment, electrochemical processes, sol-gel, biochemical treatment), and physical methods (thermal or plasma treatment, coatings), or by their combination. Table 1 contains an overview of the commonly applied methods to modify titanium surface structures into micro- or nano- scale for applications in the field of cranio and maxillofacial surgery.

The mechanical techniques, like grinding, polishing, machining, or blasting, have the main objective to obtain specific surface topographies designed to improve the adhesion of bioactive molecules and to accelerate the biomineralization as a result of increased surface area. There are some disadvantages in the case of metallic materials, as machining usually produces deformations, while blasting and grinding procedures can induce abrasive pollution on the implant surface [31]. While the classical machining technologies, grinding and polishing are used to remove the contaminated surface layers and to obtain a low surface roughness, and sandblasting is used with the aim to obtain a rough surface with functional topographies, depending on the pressure of the air jet and the particles morphology. For this reason, these classical methods are rarely solely used, due to the limited improvements in terms of surface properties.

The attrition technique is an alternative to fabricate nanophase surface layers on titanium of commercial purity which improve the tensile properties and surface hardness, rough morphology, and higher hydrophilicity [24,31]. As an evolutionary technology from sandblasting, shot peening can be used for surface-modified layers with refined grains at about 25–80 nm, with improved corrosion resistance [34]. Friction stir processing and attrition treatment are also advanced mechanical technologies used in order to obtain ultra-fine Ti grains with better biological affinity compared with coarse Ti grains, in a simple and economic way. On the other hand, conventional physical methods require expensive equipment that can produce a flame or plasma arc with high-speed gas flow and temperature between 1700 and 2700 °C (flame) in order to produce uniform coatings of hydroxyapatite, CaO–SiO_2_, CaO–MgO–2SiO_2_, TiO_2_, and Al_2_O_3_ [24,34].

The plasma polymerization technique is an alternative method used to produce a bioactive surface on Ti substrates with the aim of the better immobilization of bioactive molecules, such as DNA, heparin, and glucose oxidase [48]. NH_2_ and COOH based plasma polymers are most commonly used since these groups are known for their good chemical reactivity, or alternatively used for bacterial adhesion and biofilm prevention by coating the substrates with a suitable antibacterial agent (AgNPs). However, their aging and stability remain major issues for biological applications [48] along with the possibility of cracks occurrence during the remelting and solidification on the surface and low bond strength of the bioceramics coatings with substrates [34]. In order to produce a nano-scale texture on a titanium surface, physical vapor deposition techniques (including evaporation plating, ion plating, sputtering) alone or in combination may offer a variety of textures suitable for medical applications, but the best results were reported for plasma/ion implantation and deposition [24,34]. Even if sputtering is preferred for medical applications, due to its flexibility and ease of use, the physical methods and techniques are generally considered expensive, requiring complex systems and very high qualified technicians. The advanced physical techniques are continuously improved, reaching 1 nm ~10 nm of the surface modified layer, for improved wear resistance, corrosion resistance, and blood compatibility. In comparison with physical methods, chemical and electrochemical treatments are more affordable, being produced based on redox reactions.

Chemical methods include acidic or alkaline treatments [32,62], hydrogen peroxide treatment [63], chemical vapour deposition [64], electrochemical techniques (anodic oxidation) [65,66,67], and biochemical techniques for biomimetic coatings [68,69,70,71,72]. These techniques are able to produce specific surface topographies for improved corrosion resistance, improved biocompatibility, bioactivity, or bone conductivity. By tailoring the anodization conditions, different nanostructures can be obtained in terms of nanotubes, nanopores with a hole morphology, or nanochannels with different diameters from 15 nm up to 300 nm and different lengths, as Roy et al. [70] reviewed, pointing to the mechanisms involved in anodization and recent advances in the formation of nanostructured titanium surfaces. Oxide films prepared on Ti and Ti alloys by chemical and electrochemical treatments show complicated surface morphologies with nanostructures, among which the composite coatings proved their versatility due to their multiple functions or better properties [34]. For example, sol-gel deposition ensures the control of the purity and chemical composition of highly homogeneous films using simpler equipment and at lower cost compared to physical techniques. For biomedical applications, calcium and phosphate ions can be incorporated into nano sized anodic oxide films using an electrochemical setup. Moreover, by tailoring the anodization conditions, TiO_2_ nanotubes with a diameter ranging from 15 to 300 nm can be obtained on the surface, with favorable properties, such as better adhesion, proliferation, ALP (alkaline phosphatase) activity, and bone matrix deposition [54,55].

The available biochemical techniques for treatment of titanium implant surface are: (i) physico-chemical adsorption, (ii) covalent binding of a specific biomolecule, and (iii) peptide inclusion into a carrier material. The specific cells response can be tailored by the means of surface immobilized peptides, proteins, or growth factors, but the main drawback remains in terms of not ensuring a controlled deposition. Moreover, with the Ti substrates, being bioinert in nature, a supplementary treatment is necessary before immobilization of peptides or proteins, such as plasma polymerization, in order to activate the titanium surface [34]. Also, antibiotics and or/growth factors can be incorporated into the surface layer by applying a combination of surface treatments to obtain multifunctional coatings for biomedical applications.

### 2.2. Improved Biological Properties of Nano-Structured Surfaces

In terms of mechanical properties, the surface modifications summarized in Table 1 will result in important achievements, such as improved wear, fatigue, and corrosion resistance. It is well known that the wear resistance of coatings materials is basically determined by the microhardness and elastic modulus. On the other hand, corrosion is one of the critical issues in biomaterials science. When in contact with biological fluids, titanium metallic atoms are transformed into cations, and develop a passive film by reacting with OH^−^ and, hence, act as natural corrosion protection of the underlying substrate. An appropriate surface modification is aimed for to retain the natural properties of underlying bulk Ti substrates as well as to improve their anti-corrosion potential.

Besides the wear and corrosion resistance improvement upon different surface treatments, the expected bioactivity should be taken into account when selecting an appropriate surface modification technique. The prediction of the bone response to a nanostructured titanium surface is very difficult and many factors have to be taken into consideration, as the osseous cellular interactions at the implant interface are complex process. Bones have multiple and various functions, and hence the mechanism underlying bone biomaterial-mediated osteogenesis is conducted with the cooperative participation of the host immune cells, the host bone cells, and the material itself, being directly correlated to the biochemical and biomechanical characteristics.

**Figure 3:**
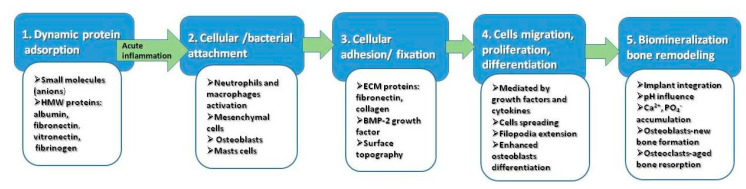
The main sequence of events occurring in vivo, during interaction between Ti surface and biological environment.

It has been demonstrated that topographical features of the implant surface are the determinant factor with respect to the cellular response [73,74]. Bone is a hierarchically structured material and highly anisotropic. The bone tissue itself consist of nanoscale architecture: osteocytes (bone-forming cells), collagen (type I) fibrils (the organic matrix), and hydroxyapatite (the inorganic bone component) are all nano-scaled structures in the range of 50–300 nm length and 0.5–5 nm width [75]. The network of collagen-crystal nano-arrangement represents the main factor influencing the mechanical properties [75]. There is a physiological, natural phenomenon, called bone remodeling, in which a continuous process of bone formation and degradation is balanced, in a very complex process regulated by growth factors and cytokines [76]. Although the detailed osteogenesis mechanisms at the bone–implant interface are still unknown, the general consensus is that the first response upon the contact with titanium implant is wetting followed by rapid protein adsorption to its surface, depending on physicochemical characteristics (pH, temperature, hydrophobicity, protein structure). Five distinct stages are assumed to occur in vivo, during the interaction between Ti surface and biological milieu [24], as represented Figure 3.

In the initial stage, the negative charged small molecules are attracted by the positive charge of titanium, followed by high molecular weight molecules with functional groups. This is a dynamic stage, involving the physical adsorption of fibronectin, albumin, fibrinogen, and immunoglobulin IgG. However, the in vivo competitive adsorption processes and their kinetics remain unclear. Initial acute inflammatory reaction may occur at the final of this stage, by activation of neutrophils. In the second stage, the nanostructured surface created by competitive protein adsorption, will promote both cellular and bacterial attachment. The crucial factor for the initiation of osseointegration is the involvement of healing macrophages in this stage. In the third stage, the attached cells are further fixed by extracellular matrix anchoring proteins. Integrins acts as a primary receptor in this stage. Mesenchymal cells, neutrophils, and macrophages are collectively involved releasing cytokines and growth factors, which subsequently interact with fibroblasts and osteoblasts. Proliferation and cell differentiation mediated by cytokines and specific growth factors occurs in the fourth stage. Enhanced osteoblasts spreading and filopodial extension are characteristics on nanorough titanium surface [38,49]. Finally, in the fifth stage, the biomineralization and osteointegration of the titanium implant occurs as a result of local changes of the pH and accumulation of Ca^2+^ and PO^4−^. At this final level, the balance between osteogenesis and osteoclastogenesis is shifted towards the last one, the osteoclasts being involved in aged bone resorption [77]. Based upon these findings, it is largely accepted that tailoring the surface chemistry of implants opens the possibility to transform osteoconductive materials into osteoproductive ones. Therefore, the new trend in titanium cranioplasty is oriented and designed to modulate the immunological response while promoting the osseointegration.

## 3. Types of Nanoparticles Used for Surface Modification of Titanium Implants

Nowadays, it remains a challenge on the topic addressed to titanium surface modification, and the debates are related to the fabrication methods for a nanostructured, porous titanium surface with effective biological activity, using facile, eco-friendly, and cost-effective approaches. A novel strategy to improve the success of soft and hard tissue integration of titanium implants is the use of nanoparticles coatings made from basically any type of biocompatible substance, which can advantageously enhance the properties of the material, as compared to its similar bulk material [78]. Nanotechnology has enabled the addition of metals into their nanosize, leading to extreme changes in chemical, physical, and optical properties of metals, as the metallic nanoparticles are the most promising agents known for enhanced antibacterial activity. By these techniques, implant surfaces should be designed not only to support the attachment of target tissue cells, but also to prevent bacterial adhesion. One main strategy to decrease bacterial infiltration is to limit biofilm formation due to the initial contamination of the implant surface. Biofilms consist of microorganism aggregates adherent to the implant surface, comprising multiple species with high cell densities, which critically influence the implants function and may initiate local inflammation [79]. So, most of the physical methods approaches involve compaction of nanoparticles versus micron-level particles to yield surfaces with nanoscale grain boundaries, preserving in the same time the chemistry of the surface among different topographies. Based on literature research, in Table 2 are summarized the types of nanoparticles used for surface modifications of titanium implants, the fabrication methods, the main features, and the purpose of use.

**Figure 4:**
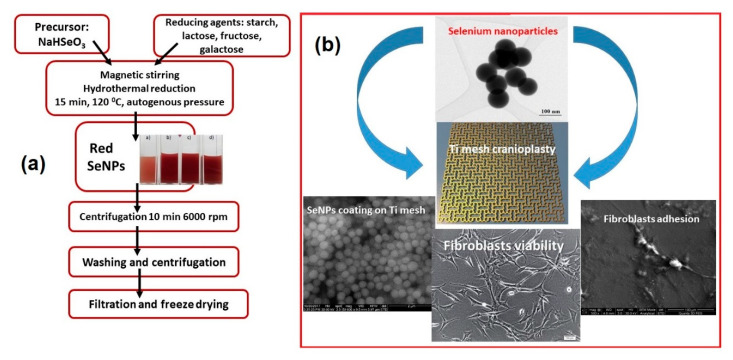
(**a**) The flow chart of SeNPs production via hydrothermal reaction using different saccharides as reducing agent; (**b**) TEM image of SeNPs used for the surface modifications of Ti mesh for cranioplasty, along with the surface morphology of the coating upon in situ SeNPs deposition and details of fibroblasts adhesion on the nanostructured Ti surface.

As an alternative to antibiotics, nanoparticles are known as one of the most effective antibacterial agents, and nowadays, surface modification of titanium using antibacterial properties of metal nanoparticles is a popular approach in clinical treatments. Antimicrobial nanoparticles used for decorating the titanium surfaces mainly include Ag, Zn, Au, and Se [89,90,91,92,93], but also less investigated antimicrobial agents such as copper, fluorine, and calcium [83,95,96]. In a recent study, Esfandiari et al. [96] prepared Ag-decorated TiO_2_ nanotubes by a combined electrochemical and UV-assisted reduction method, showing a synergetic bactericidal effect of ~100 nm TiO_2_ nanotubes and 20 nm Ag NPs. Besides the antimicrobial effect, nanostructured titanium surfaces (created with different nanoparticles) demonstrated better implant fixation. Moreover, many efforts have been made to create nano-structured roughness to simultaneously increase healthy bone growth while inhibiting cancerous bone growth [93,94,95,97]. As selenium is a well-known anti-cancer chemical, nano-rough selenium was developed for orthopedic applications, including cranioplasty, involving bone cancer treatment [92,93]. In a comparative study performed by Tran and col. [94], the authors demonstrated a superior adhesion of healthy osteoblast on nano-rough selenium compacts versus the micro-rough ones. They proposed a mechanism in which the initial absorption of proteins (such as fibronectin and vitronectin) was favored by the nano-topography, followed by subsequent cell adhesion. Selenium nanoclusters were shown to promote healthy osteoblast functions after one day of culture, and more importantly, to inhibit cancerous osteoblast cell functions after three days of culture [94]. Recently, our research group proposed an improvement of the surfaces [92,93], in the case of titanium mesh for cranioplasty by the in-situ hydrothermal deposition of selenium nanoparticles obtained using an original method (starch, glucose, and galactose were selected as reducing agents), as summarized in Figure 4a. The surface modification (Figure 4b) may offer important benefits in terms of osteointegration, without using additional screws for fixation and closure procedure. However, considering the toxicity of selenium at high levels [98], a careful dosage of selenium in compacts is necessary in order to avoid unnecessary exposure and possible toxic effects.

Osteoblast adhesion on Ti surfaces is a prerequisite for successful implant integration, as demonstrated by in vitro studies devoted to interaction between osteoblasts and nano-structured Ti surface. Webster et al. provided the first evidence of increased osteoblasts adhesion on nanophase Ti compared with conventional one [99]. The explanation was related to the increased surface area and nanoroughness which generated a better wettability and, consequently, promoted the adsorption of hydrophilic proteins (e.g., vitronectin, fibronectin) involved in bone cell attachment. Later studies demonstrated that osteoblast adhesion significantly increased by 33% on anodized Ti compared with conventional Ti, accompanied by an increase in fibronectin and vitronectin adsorption [100]. With respect to the size and morphology of the surface, Oh et al. [101] demonstrated that nanotube diameter regulates stem cell differentiation. Smaller nanotubes (~30 nm) are involved in mesenchymal stem cell adhesion, while larger nanotubes (~70–100 nm) are involved in cellular elongation and differentiation into osteoblast-like cells. Taking into account the scale-range of bone matrix represented by osteocytes, hydroxyapatite crystals, and collagen fibrils, respectively, 50–300 nm in length and 0.5–5 nm in width, the designed implant surfaces must fit the physiological milieu and mimic the growing conditions for osteogenic cells [24].

There are many controversies in the literature with respect to the effect of nanoscale topographic details of titanium implants. It was suggested that a minimal 100 μm pore size is required for bone tissue regeneration, while a smaller pore sized remains prone to fibrous tissue formation [84,102]. Clinical and in vivo information about these nano-size coatings and their beneficial applications is still rare. In 1983, Branemark et al. [103] described the osseointegration as a direct structural and functional bone to implant contact under functional load. Various animal models were performed in order to study the enhanced osseointegration by surface treatments at the nanometer scale. Kubo et al. observed a significant increase in bone-titanium interfacial strength after two weeks of implantation in femur rats using Ti nanotube (300 nm) surface modification [104], while in a similar in vivo experiment, Ogawa et al. [105] demonstrated that an increased surface area up to 40% was correlated to higher osseointegration of nanostructured Ti compared to an acid-etched surface. A very recent in vivo study of plasma-sprayed carbon nanotube (CNT)-reinforced hydroxyapatite (HA) coating on titanium implants embedded in rodents’ bone demonstrated that CNT addition induces higher osseointegration as compared to HA [106]. In this case, another important evidence of implant mechanical integrity was proven by the similar value of elastic modulus of the newly grown bone compared with the distant bone. In another study, a 40–80-nm thin layer of gold nanoparticles was deposited by the magnetron sputtering technique on a porous Ti surface [91] and inserted in the proximal region of the humerus in canines. This study suggests that early biomechanical fixation was influenced by the gold nanostructured coating, pointing out that released gold ions are able to reduce the inflammatory process. However, the authors concluded that a long-term study is necessary to highlight the anticipated anti-inflammatory effects of the coating. A multi-database systematic literature review was conducted by Bral and Mommaerts [106] with the objective to provide a structural review of the fabrication techniques to produce a CaP coating with good in vivo results compared to micro-CaP–coated and uncoated titanium implants. This study was elaborated based on twenty-eight papers consisting of both animal models and human studies. Once again, it was demonstrated that titanium implants coated with nano CaP and nano-hydroxyapatite improves implant fixation, based on histomorphometrical healing properties. They also concluded that not all coating techniques have beneficial effects on biofunctionalization. Several factors might have a crucial importance in successful biofunctionalization: implant nano-roughness, coating thickness, calcium phosphate solubility, and nanotopography. However, the nano-HA pro-mimic coating has exhibited good results in vivo, in an animal model and in human studies involving early and intermediate healing periods [106]. Either chemical or physical deposition techniques proved to be effective methods for producing versatile and biocompatible nanostructured coatings with different chemical and morphological properties to promote a better bone tissue response. By tailoring the surface of titanium implants for cranioplasty and related fields, the results may contribute to the general efforts dedicated to continuous improvement of nano-biomaterials, opening new possibilities for long-term development and strategies in nanomedicine [93,107].

## 4. Patented Solutions to Improve Osteointegration of Cranial Implants

### 4.1. Cranial Endoprosthesis with Sliding System

The invention relates to a cranial stent with a sliding system, used for the repair of traumatic defects of the skull, through the cranioplasty surgical procedure.

Currently, the most used medical devices for cranioplasty are: plaques, nets, distractors, autografts and allografts [107,108], fastened by screw and mini-plates. The fixation is made in relation to the contour of the uninjured skull. The disadvantages of the above-mentioned solutions, known from the prior art, are related to the need for customization with low cost and in the same time avoiding additional drilling of the skull. The additive manufacturing systems evolved in parallel to the novel medical devices and nanotechnologies in order to develop new constructive forms as close as possible to the patient’s anatomy. Due to its advantages, the manufacture of custom implants is currently considered to bring the highest level of benefits in terms of compliance with patient needs [106]. Also, a personalized implant significantly reduces the duration of surgery and, implicitly, postoperative complications. However, a cranial implant made by single production has high costs compared to standardized devices on the market [108,109,110].

**Figure 5:**
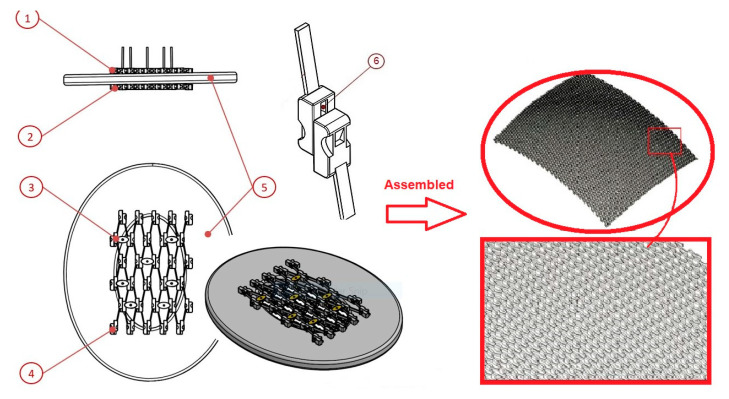
Left panel- Components of the cranial endoprosthesis with sliding system: Upper sliding layer ①; lower sliding layer ②; fixing system ③. The sliding layers ① and ② are composed of multiple mobile cells with sliding system ④; Positioning of the stent in relation to a schematic model of the cranial box ⑤; Conjugate sliding system ⑥. Right panel-The assembly of the mobile cells to obtain constructive anatomical curvature in order to facilitate customization.

We propose one innovative solution that supposes the adaptation to the customized dimensions of a patient without the need to process specific anatomical data and the manufacture of a unique cranial prosthesis. The cranial prosthesis, with a sliding system that consists of an upper sliding layer, a lower sliding layer, and a fixing system, eliminates the mentioned disadvantages, as the sliding system can be manufactured in several types of dimensions for the repair of cranial defects of different sizes and shapes (Figure 5). Also, the newly proposed solution solves the problem of complications arising from the perforation of the cranial box when fixing classic implants. The fixation in the case of cranial endoprosthesis with a sliding system is achieved by the constructive form of the stent, through a fixing system with clamps.

The main advantage of the newly proposed cranial prosthesis with a sliding system consists in the adaptation of the prosthesis to various dimensions of the cranial defect, hence facilitating the serial production while maintaining the character of customization according to the patient’s anatomy. The fixation system does not require drilling the patient’s skull. In this way, post-traumatic complications are avoided, the duration of fixation is minimized, and consequently, the duration of entire intraoperatively procedure is also reduced, promoting a faster patient recovery.

The cranial prosthesis with sliding system consists of an upper sliding layer, a lower sliding layer and a fixing system, the sliding layers being composed of mobile cells (they have a general parallelepiped shape with connected edges, but can have any other shape that allows for the execution of the sliding movement, such as: spherical, cylindrical, prismatic, pyramidal, etc.) with the sliding system. For assembly, the lower sliding layer is positioned in a non-slided state, tangential to the lower surface of the skull, while for sliding and engaging the moving cells in the lower layer, an actuating key is required. On the side of the movable cells, the conjugate gear surfaces are provided, which come into contact with the actuating key for engaging the sliding movement. The upper and lower layers of the mobile cells are provided with slots for the orientation and positioning of the fastening system. The upper and lower layer has a conjugate surface for laying and orienting. The sliding movement is performed by means of the sliding and conjugate sliding surface, the movement being transmitted from one cell to another, propagating the sliding movement.

The assembly method of the mobile cells is shown in Figure 5 for a cranial prosthesis with overall dimensions between 80 mm and 120 mm. The sliding layers of the stent have a constructive anatomical curvature in three planes to facilitate customization according to the patient’s anatomy and the shape of the traumatic hole.

### 4.2. Titanium Mesh with Novel Geometry and Functional Nano-Coating Based on SeNPs

According to the literature, complications of cranioplasties can be classified into three main categories: (1) septic complications; (2) neurological complications; (3) cerebrospinal fluid complications [110,111,112,113,114,115,116]. Infections and related complications may become dramatic, requiring special management with additional economic costs and prolonged hospitalization. It has been demonstrated that the timing of cranioplasty, the size of the defect, and the biomaterial choice may also influence the subsequent planning and post-operative management to reduce all types of complications [117,118,119,120].

In this context, the proposed solution relates to a structure surface and method for fixing a mesh-type cranial prosthesis, executable with biocompatible titanium alloys, having functional coatings based on selenium nanoparticles with osseointegration properties, used for cranioplasty and reconfigurations of major cranial defects caused by accidents, birth defects, and surgery due to tumor extractions or other cranial diseases. The disadvantages of titanium implants for cranioplasty are represented by the fixing system with titanium screws, as well as the absence of osseointegration, due to the inert character of titanium and its alloys in contact with human hard tissues.

The advantages of the cranial implant with osteointegration structures and functional coatings are based on mimicry, related to the basal cells with a spider web geometric structure, and geometric elements characterized by sharp angles, which favors the capillarity process for long-term maintenance [121,122,123]. Moreover, the particles deposited on the surface will promote osseointegration allowing for the fast restoration of human ordeal. Se nanoparticles are produced by eco-friendly processes, avoiding the use of harsh, toxic, and expensive chemicals [124]. Also, the system of alternating rigid and flexible bridges allows for the adaptation of the cranial implant with osseointegration structures to the patient’s anatomy, maintaining the necessary mechanical strength characteristics.

**Figure 6:**
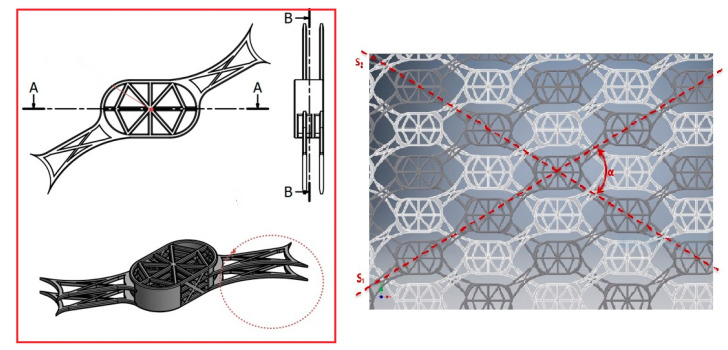
The components of the modular system. Left panel-stratified basal cells, the rigid and flexible connectors. Right panel-the arrangement of modular cells in alternately configuration, so that all rigid connecting bridges align in the S_1_ direction, while the flexible ones align in the S_2_ direction.

As presented in Figure 6, the first constructive form of the osseointegration module consists of stratified basal cells and two rigid bridges arranged symmetrically on the diagonal of the osteointegration module. Basal cells are connected by a resistance structure. The same resistance structure connects all four basal cells, over the entire height of the osseointegration module. The rigid bridge consists of two connecting units and two resistance pillars with a semicircular contour. The resistance pillar is arranged symmetrically on the diagonal of the module, on the entire height of the osseointegration module, connecting all four basal cells. The connecting units, which together form the rigid bridge, are arranged alternately on the first and third layers of the basal cells in the osseointegration module.

The second constructive form of the osseointegration module consists of the stratified arranged basal cells and two flexible connecting bridges arranged symmetrically on the diagonal opposite to the one containing the rigid bridges of the first constructive form. The two constructive forms of the osseointegration module have similar overall dimensions. The basal cells are connected by the resistance structure provided over the entire height of the osseointegration module. The flexible connecting bridge consists of two connecting units connected to the basic units in the module construction. The first connecting unit is connected to the basal cell arranged on the second layer, and the second unit to the basal cell arranged on the fourth layer, alternately to the connecting units in the rigid connecting bridge. The two constructive forms of the osseointegration module are alternately arranged so that all rigid connecting bridges align in the S_1_ direction and the flexible connecting bridges align in the S_2_ direction. The two directions intersect on the longitudinal axis of the implant forming the angle α.

**Figure 7:**
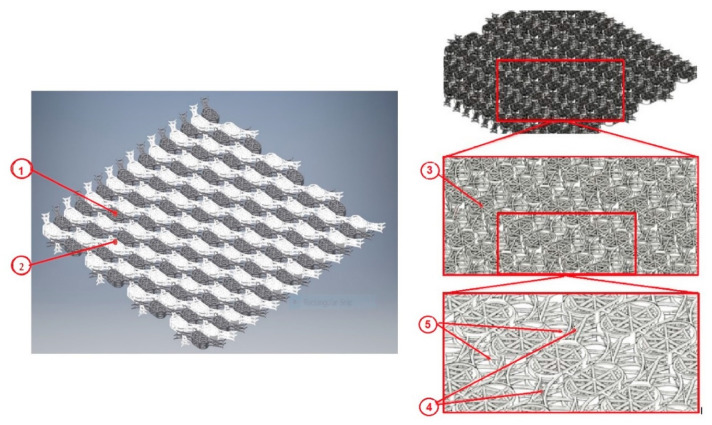
Interconnected layers of the modular system. Left panel-The fixed layer ① is assembled with the movable layer ② so that translations can be performed in the Ox and Oy directions, while maintaining the position of the layer ①. Right panel-The component elements of the cranial prosthesis presented in isometric view, consisting of four basal cells defined in two constructive forms ③, the rigid connecting bridge ④ and the flexible connecting bridge ⑤.

The cranial prosthesis with osseointegration structures, according to Figure 7, consists of the osseointegration module ③, the rigid connecting bridge ④, and the flexible connecting bridge ⑤. The osseointegration module ③ consists of four basal cells and is defined in two constructive forms.

In height, the osseointegration module has dimensions between 1 mm and 2 mm, its width being between 2.5 mm and 3.5 mm and its length between 4 mm and 5 mm. The basal cells have the same values for length and width as the osseointegration module, and the layer thickness is between 0.1 mm and 0.2 mm.

As a manufacturing technique, the prosthesis is made in one piece, in an already assembled form. Primary post-processing involves detaching from the workpiece table and deburring the support structures. The upper and lower surfaces of each osseointegration module are post-processed with superfinishing processes and cleaned by pickling and in an ultrasonic bath. Subsequently, it is impregnated with Se nanoparticles, which accelerates the regeneration process of bone tissue, improving the degree of osseointegration of the cranial prosthesis with the surrounding anatomical bone structures, as well as the antibacterial effect.

The main clinical outcome of this novel approach is represented by providing solutions in particular cases in which the size of the cranial defect is unknown preoperatively or if the alteration of an existing skull defect is required, such as craniectomy performed to remove bony tumors or osteomyelitic bone [21,113]. In these cases, prefabricated implants cannot be used. The overall clinical benefit is reflected in saving clinical time, as a decreased surgical timeframe also means a decrease of blood loss and anesthesia exposure time. On the other hand, tailoring the titanium surface with functional nanoparticles offers important clinical applications due to simultaneous antibacterial and osteoconductive activities in patients with bone healing disorders associated with rheumatoid arthritis, osteoporosis, diabetes, or aging [25,94,96,106].

## 5. Conclusions and Future Perspective

Although autologous bone seems to be the standard material with the best biocompatibility properties, being also osteoconductive and osteoinductive, it has several limitations: only certain parts of the human body can provide an ideal bone graft, being difficult to model intraoperatively because it does not allow perfect coverage of the intracranial bone defect. For this reason, advance research and development of alloplastic biomaterials has become an important goal of materials science.

One can observe that, over time, there has been no ideal biomaterial developed for cranial prosthesis used in cranioplasty. However, biomaterials with high mechanical strength, resistant to infections, radiolucent, cheap, easy to handle and able to reintegrate into the skull, are the most used materials in cranioplasty. Each alloplastic biomaterial used to manufacture cranial prostheses has both advantages and disadvantages. It is important that the craniofacial surgeon, together with the medical engineer, can make the most appropriate choice, taking into account the anatomical and physiological characteristics of each patient and the type of trauma. Surgical replacement of bone tissue with a synthetic material has both a protective and an aesthetic role, which is why it is so difficult to choose a particular material for reconstruction. Although many different methods have been described in the literature, using autologous or alloplastic material, there is still no consensus on which method is better, and continuous multidisciplinary researches are conducted aiming to develop the ideal reconstruction materials.

This review emphasized the main reasons why titanium mesh is preferred for skull reconstructions along with the importance of developing innovative surface structures with a dual benefit in terms of improved osteointegration and enhanced antibacterial activity to reduce the risk of post-surgical infection, knowing that infections are the main complication in cranioplasty surgeries. Based on literature research, the identification of the nanoparticle types designed for the surface modification of titanium implants was conducted, along with the fabrication methods, their main features, advantages, and disadvantages with respect to their purpose of use.

It has been found that performing cranioplasty with titanium mesh prostheses has decreased infection rates in high-risk patients, such as military personnel who have suffered extensive scalp injuries and/or craniectomy surgeries during the war in Iraq and Afghanistan. In addition, computer-aided 3D modeling was used successfully to design titanium mesh prostheses which provide an excellent cosmetic effect, even in the case of large cranial defects.

Using the specialized software Autodesk Inventor Professional 2016, a novel intracranial prosthesis was designed. The design is a mesh type which does not require fixing to the skull with screws. The aim was to create a sliding system which allows for the adaptation of the cranial prosthesis to variable dimensions of bone deficiency, respecting the patient’s anatomy, without the need for other fastening systems (screws, wires, etc.) that would cause additional trauma or prolonged surgery time. Different types of coatings (calcium phosphate, hydroxyapatite) can be used for this intracranial prosthesis, with the role of facilitating and accelerating the osseointegration process.

To promote the osseointegration process, the second type of intracranial prosthesis has been designed, whose constructive elements have a porous microstructure that promotes the absorption of osseointegration particles (Se nanoparticles) and promotes bone tissue regeneration. Selenium nanoparticles were proposed for developing the coatings with an osseointegration role, being fabricated by an eco-friendly method. Thus, the NaHSeO₃ salt (sodium hydrogen selenite) was used as a precursor, and molecules from the category of carbohydrates were used as reducing agents: starch (polysaccharide), lactose (disaccharide), galactose and fructose (monosaccharides) [124,125,126,127,128,129,130].

The future in cranioplasty is related to molecular biology, which involves the application of bone growth factors to help and stimulate bone formation in prosthesis. So, currently research related to cranioplasty is pursuing a new approach. The mechanism by which undifferentiated mesenchymal cells can be transformed into osteoprogenitor cells is being investigated, in order to favor the osseointegration of intracranial prostheses. Making an intracranial prosthesis with osseointegration properties is a complicated process that involves revascularization and bone formation in the prosthesis. The ideal prosthesis should function as a scaffold for the progressive proliferation of blood vessels and osteoprogenitor cells.

Through the osteoinduction process, osteoprogenitor cells should not migrate from the surrounding tissue. Instead, they will be produced “in situ” with the help of bone morphogenetic proteins, and then infused into a prosthesis structure. These bone growth factors could be integrated into sustained-release polymers to stimulate cellular responses to bone regeneration, providing immediate protection of the skull, with aesthetic benefits along the osteoconductive and osteoinductive advantages.

Also, the future belongs to the nanotechnology design, obtaining nanometric particles that can be used in medicine to cover different types of prostheses, so that the process of osseointegration is favored. Our work is aligned to the general efforts dedicated to continuous improvement in the field on nano-bio-materials, opening new possibilities for long-term development and strategies in nanomedicine, including antibacterial, osteoconductive, and osteoinductive nanomaterials, which may offer the desired biological response.

## 6. Patent

OSIM nr. 132417, release date 30/10/2019. Title: Cranial implant with osteointegrating structures and functional surface.

## Figures and Tables

**Table 1:** Mechanical, physical and chemical methods used for surface modification of Ti implants for cranial and maxilofacial surgery.

**Mechanical Methods**		
**Technologies**	**Texture/Roughness Size**	**Outcome/Reference**
Machining Grinding Blasting	~1 μm, rough surface formed by subtraction process	Specific surface topographies. Improved adhesion and bonding. Auxiliary method to remove contamination. Rarely solely used [25,26,27,28,29,30,31,32]
Shoot peening	20–80 nm grains on the surface	Improved fatigue resistance, hardness and wear [33,34]
Friction stir processing (FSP)	<1 μm, ultrafine grained surface	Improved sliding friction and wear resistance. Incorporation of AgNPs, Zn with antibacterial effect [34,35]
Attrition	<100 nm grains on the surface	Improved tensile properties and surface hardness, higher hydrophilicity, better biological affinity [24,36,37]
Hydrothermal & pressure (HPT)	flake-like titanate layer on Ti substrate, pore size of 300–600 nm	Minimize the time-consumption and the manufacturing cost. Enhance the in vitro cell-material interactions [38]
**Physical Methods**		
**Technologies**	**Texture/Roughness Size**	**Outcome/Reference**
Thermal (flame or plasma) spraying	~30 to ~200 μm of coatings, such as TiO_2_, HA, CaP, Al_2_O_3_, ZrO_2_, TiO_2_	Improved wear/corrosion resistance and biocompatibility [24,39,40,41]
Physical vapor deposition: evaporation, sputtering, ion plating	<1 μm, TiN, TiC, TiCN, TiO_2_, amorphous carbon films, full density	Improved wear/corrosion resistance and blood compatibility [34,36,42,43]
Ion implantation and deposition	~10 nm of surface modified layer and/or thin film such	Improved hardness, wear, fatigue/corrosion resistance
	as Ti–O, Ti–N films	and blood compatibility [44,45]
Plasma treatment	<100 nm, TiO_2_, TiN, TiOH, TiCN layers, full density	Clean and sterilize surface, remove native oxide layer. Improved hardness, wear and corrosion resistances, fatigue limit and biocompatibility [46,47]
Plasma polymerization	Not reported	Bioactive surface. Improved cell adhesion [48]
**Chemical Methods**		
**Technologies**	**Texture/Roughness Size**	**Outcome/Reference**
Acidic treatment (HF, HCl, H_2_SO_4_)	~10 nm oxide layer on the surface	Remove oxide scales and contamination. Used in combination with other treatments (blasting), higher roughness promoting osteoblasts attachment [49,50]
Alkali treatment (NaOH, KOH)	~1 μm sodium titanate gel on the surface	Improved biocompatibility, bioactivity or bone conductivity [32,51]
Hydrogen peroxide treatment	Inner oxide layer <10 nm; outer porous oxide layer up to 40 nm	Improved biocompatibility or bioactivity [34,52]
Passivation treatment (nitric acid, phosphoric acid)	~2–30 nm oxide layer dominated by TiO_2_, uniform, full density	Enhanced corrosion, resistance and wear resistance, better bioactivity compared to mechanical treatment [34,53]
Electrochemical methods (anodization, electrodeposition)	~10 nm–10 μm uniform, controllable thickness of TiO_2_ layer; adsorption and incorporation of electrolyte anions	Improved adhesive bonding, corrosion resistance, bioactivity, specific surface topographies [54,55]
Chemical vapor deposition	~1 μm of TiN, TiC, TiCN, diamond and diamond-like carbon thin film, nearly full density	Extremely high hardness and wear resistance compared with Ti substrate. Improved corrosion resistance and blood compatibility [56,57]
Sol-gel	<10 μm of thin ceramic coatings, such as Ca_3_(PO_4_)_2_, TiO_2_, SiO_2_	Highly homogeneity and improvement in bioactivity [58,59]
Biochemical methods (by soaking- peptide, proteins immobilization, functional molecules, drug loaded)	self-assembled monolayers, does not ensure controlled deposition	Improved bioactivity, biocompatibility, and/or antibacterial functions [60,61]

**Table 2:** Nanoparticles used for surface modification of Ti implants.

Type of NPs	Fabrication Method	Main Features/Purpose	Reference
TiO_2_	direct oxidation	Nanofibers, nanoneedles/better hydrophilicity, biocompatibility and antimicrobial activity, compared to Ti6Al4V	[80]
	Pulsed laser deposition	Nanorods/Nontoxicity, ability to increase the density of osteoblast cells on the implant, enhanced osseointegration, anticandidal effect, bone formation ability	[81]
	Anodic oxidation	Nanotubes incorporating Ca, P and Ag/cells migration on the Ti-based implants due to super hydrophilic properties of crystalline TiO_2_ nanotubes; apatite formation in simulated body fluid, enhanced MC3T3-E1 cell adhesion and proliferation, antibacterial effect against S. aureus.	[82,83]
	plasma electrolytic oxidation	Nanostructured Zn-incorporated TiO_2_ coatings, grains 20–100 nm/inhibition of *S. aureus* and *E. coli*	[84]
Al_3_O_3_	micro-arc oxidation	Nanostructured surface for improved microhardness and wear resistance	[81,85]
	dipping	Nanostructured surface/promote MSC commitment to	[86]
	-	the osteoblast phenotype, increase in bone-implant contact area and torque removal	-
Nano Hydroxyapatite	discrete crystalline deposition	Complex surface morphology via the bonded HA nanoparticles/progressive osseointegration profiles	[78,87]
Nano-crystalline diamond	Plasma spray	Nanosized crystallites/proteins immobilization on nanocrystalline diamond/osteoinductive effect in irradiated bone	[78,88]
Ag NPs	silanization method	Spherical morphology, 100 nm diameter, antibacterial and anti-adhesive activities towards *S. aureus* and *E. coli.*	[24,83,89]
ZnO NPs	EHDA spraying.	rod-shaped structure ~100 nm/significant antimicrobial activity against Staphylococcus aureus/early bone formation	[90]
Au Nps	Magnetron sputtering	40–80 nm thin layer of pure gold/early mechanical fixation	[91]
Se Nps	Hydrothermal deposition	Spherical, rods, wire nanostructure, using different saccharides as reducing agent/favorable results on RBC osmotic fragility and fibroblasts adhesion to accelerate osseointegration, bone cancer treatment.	[92,93,94,95]
